# Numerical Simulation of Optically-Induced Dielectrophoresis Using a Voltage-Transformation-Ratio Model

**DOI:** 10.3390/s130201965

**Published:** 2013-02-04

**Authors:** Shih-Hsun Hung, Sheng-Chieh Huang, Gwo-Bin Lee

**Affiliations:** 1 Department of Engineering Science, National Cheng Kung University, Tainan 701, Taiwan; E-Mail: intel_once@hotmail.com; 2 Department of Power Mechanical Engineering, National Tsing Hua University, Hsinchu 300, Taiwan; E-Mail: xaviersty@hotmail.com

**Keywords:** optically-induced dielectrophoresis, numerical simulation, emulsion droplets, polystyrene beads

## Abstract

Optically-induced dielectrophoresis (ODEP) has been extensively used for the manipulation and separation of cells, beads and micro-droplets in microfluidic devices. With this approach, non-uniform electric fields induced by light projected on a photoconductive layer can be used to generate attractive or repulsive forces on dielectric materials. Then, moving these light patterns can be used for the manipulation of particles in the microfluidic devices. This study reports on the results from numerical simulation of the ODEP platform using a new model based on a voltage transformation ratio, which takes the effective electrical voltage into consideration. Results showed that the numerical simulation was in reasonably agreement with experimental data for the manipulation of polystyrene beads and emulsion droplets, with a coefficient of variation less than 6.2% (n = 3). The proposed model can be applied to simulations of the ODEP force and may provide a reliable tool for estimating induced dielectrophoretic forces and electric fields, which is crucial for microfluidic applications.

## 1. Introduction

Mechanical manipulators [1,2] are the most intuitive methods for manipulating micro-particles. However, these techniques are inherently invasive and difficult to scale up for parallel manipulation in a large-array format. Alternatively, microfluidics provide a non-invasive way to manipulate [3,4] and sort [5] a large number of micro-particles, by using hydrodynamic forces. However, these methods require delicate micropumps and miniature flow control systems and are challenging for addressing single particles. Other electrokinetic approaches such as electrophoresis [6,7] and electroosmosis [8] have been used to manipulate various particles and bio-molecules. However, electrophoresis requires the particles to be charged and does not act on uncharged particles, while electroosmotic flow has limited particle trapping capabilities. Alternatively, magnetic forces have also been used to manipulate various micro- and nano-scale particles [9–11]. However, these methods can only address intrinsically magnetic materials or require tagging of particles with magnetic objects.

Recently, dielectrophoresis (DEP) devices has been widely exploited to manipulate and separate dielectric particles with different sizes in a variety of applications ranging from cell trapping/sorting [12–16] to carbon nanotube separation [17] and nanowire assembly [18]. The particles can be either repelled or attracted when exposed to a non-uniform electric field. Furthermore, this mechanism has been widely used to distinguish nonviable and viable cells [19,20]. Another application of the DEP force for particle manipulation and separation is based on the use of barriers built by a DEP force field [21,22]. In addition, DEP devices have parallel manipulation capabilities if multiple DEP electrodes were properly employed. However, patterned metal electrodes usually require complex fabrication processes. Another limitation of the conventional DEP method is that the electrode patterns used for particle manipulation cannot be changed once they have been fabricated, thus reducing the flexibility of this technology. Furthermore, it is difficult to isolate a single particle of interest. Particle trapping can be also achieved by creating dynamic DEP cages via complementary metal-oxide-semiconductor (CMOS) control [23]. However, this device is limited by the spatial resolution of the CMOS circuitry and is thus currently limited to micro-scale manipulation.

Alternatively, optically-induced dielectrophoresis (ODEP) technology has been reported recently to generate the DEP force by using a projected light pattern [24]. Utilizing moving optical images generated from a digital projector or a digital scanner to form “virtual” electrodes, it can provide the required DEP force which improves the flexibility of manipulating particles/cells/droplets. By using virtual electrodes instead of using fixed metal electrodes, the spatial manipulation and separation of micro-particles have been demonstrated in the ODEP platforms with polystyrene beads [24–30], blood cells [26,28], white blood cells [24,29], Jurkat cells [29], HeLa cells [29,31], yeast cells [30], nanoparticles including semiconducting and metallic nanowires [32,33], carbon nanotubes [34], metallic spherical nanoparticles [35], and DNA [36,37]. Other functionalities such as dynamic single cell electroporation [38], cell lysis [39], optically-induced flow cytometry [40], and large-scale, dynamic patterning of nanoparticles [41], have been demonstrated recently. In addition to solid particles, the manipulation of oil-in-water emulsion droplets using the ODEP platform has also been demonstrated by the current research group [42]. Therefore, the ODEP force has shown its great potential in various microfluidics-based applications.

However, very few attempts have been made to characterize the induced ODEP forces with satisfactory accuracy. Traditionally, the magnitude of the generated ODEP forces can be roughly estimated by using a simplified force balance model, which moves the particle at its terminal velocity when the viscous drag force in fluid is balanced by the resulting ODEP force [24]. Nevertheless, the measurement of the terminal velocity by the viscous drag force is not straightforward because it is difficult to experimentally determine the terminal velocity when the particle is moving in fluid. Besides, this method is only suitable for characterizing the negative ODEP force, which repels particles away and not suitable for the positive ODEP force which attracts particles. Therefore, it is essential to explore a numerical model to calculate the ODEP force rather than using a simplified force balance model.

It has been reported that the electrical field distribution can be calculated with an assumption that the ODEP device was modeled as the resistance of the photoconductive layer in series with the resistance of the liquid layer [28]. In addition, the electrical response of a photoconductor to a laser beam was modeled as a Gaussian distribution in the conductivity of amorphous silicon (a-Si:H, photoconductive layer) [27,28,31,43,44]. However, the simplified assumption of a Gaussian distribution in the conductivity of the a-Si:H layer could not accurately model the photoconductive process (photoelectric effect) because of the complex electron and photon transmission behavior. Therefore, the calculated magnitude of the ODEP force was much higher than the experimental data. It was reported that magnitude of the gradient of electric field has to be reduced significantly so that the order of magnitude of the ODEP force can be matched between the numerical and experimental results [31]. It was also found that the trap profile which included the magnitude of the ODEP force at a specific height above the a-Si:H, matched the measured trap profiles when reduced by a correction factor. However, the correction factor was difficult to be determined systematically. Therefore, the widely-used model based on a change in the conductivity of the a-Si:H layer cannot calculate the ODEP force with satisfactory accuracy.

Moreover, a method to calculate the ODEP force exerted on a water-in-oil droplet by integrating the Maxwell stress tensor over the whole droplet surface, rather than using the DEP formula, has been reported recently [43]. However, their simulated device was different from usual ODEP devices since a high direct-current bias was applied to lateral electrodes instead of using the more common longitudinal ODEP configuration. Therefore, the ODEP force is still challenging to calculate or to simulate with a satisfactory accuracy. An equivalent electrical model [45] was recently proposed to separate the illuminated and non-illuminated photoconductive structural layers, which was equivalent to a parallel structure of resistors and capacitors. Nevertheless, the electric field distribution basically behaved like the DEP force because of the zone-separated geometries and the direct input of voltage as a step-function boundary without considering the optical process (light dispersion). The simplified assumption of uniform light propagation on the ODEP electrode may result in a significant variation of the calculated electric field and the resulting ODEP force. Furthermore, the magnitude of the ODEP force was not reported in that work to show a direct comparison between the simulated and experimental results.

**Figure 1.**
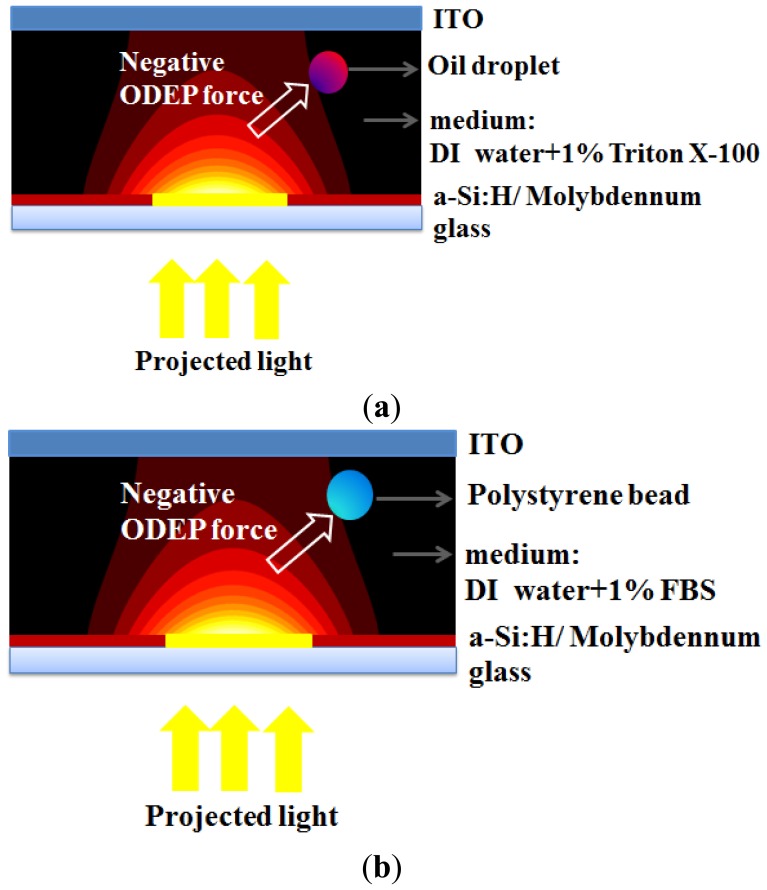
Manipulation of micro-objects in an ODEP device, including (**a**) oil-in-water emulsion droplets with deionized (DI) water and 1% Triton X-100 surfactant (**b**) polystyrene beads with DI water and 1% fetal bovine serum (FBS).

Therefore, in the current study, we propose a new “voltage-transformation-ratio” (VTR) model to numerically simulate the electric field for both emulsion droplets (Figure 1(a)) and polystyrene beads (Figure 1(b)) and then calculate the resulting ODEP forces. Results showed that our numerical model can reasonably match the experimental data. This can pave a way for calculating the ODEP forces in future biomedical applications.

## 2. Experimental and Numerical Simulation Section

### 2.1. DEP Force

The DEP force acting on a dielectric particle is caused by the interfacial polarization between the particle and the media in a non-uniform electric field [46]. For the case of a solid sphere and a droplet, the effective dipole moment (p) is expressed as follows [47].

(1) $$p=4\pi {ɛ}_{m}\left(\frac{{ɛ}_{p}^{*}-{ɛ}_{m}^{*}}{{ɛ}_{p}^{*}+2{ɛ}_{m}^{*}}\right)r^{3}E$$

(2) $${ɛ}^{*}=ɛ-j\left(\frac{\sigma }{\omega }\right)$$

where r is the radius of the particle, E is the electric field, *ε_p_* is the permittivity of the particle, *ε_m_* is the permittivity of the media, σ_m_ is the electrical conductivity of the media, σ_p_ is the electrical conductivity of the particle, ω is angular frequency, and j is the imaginary unit. The time-averaged DEP force acting on a spherical particle, immersed in a medium and exposed to a spatially non-uniform electric field can be then described as follows [46].

(3) <FDEP>=(p⋅∇)E=2πr3ɛmRe[K*(ω)]∇|Erms|2

(4) $$K*(\omega )=\frac{{ɛ}_{p}^{*}-{ɛ}_{m}^{*}}{{ɛ}_{p}^{*}+2{ɛ}_{m}^{*}}$$

where Re[K*(*ω*)] is the real part of Claussius-Mosotti (CM) factor, which can be determined by the frequency-dependent complex permittivities of the particle and medium. The CM factor is often used as an indicator that describes how the effective dipole moment of the particle varies with the material properties and the frequency of the applied voltage. It is noted that the induced ODEP forces are proportional to the gradient of the square of the electric field strength (∇*E*^2^).

### 2.2. The Simulation Cases and Experimental Setup

Figures 1(a,b) show schematic illustrations about the manipulation of oil-in-water emulsion droplets and polystyrene beads. The continuous-phase liquid (water) containing dispersed oil droplets (oil-in-water droplets) were sandwiched between these two indium-tin-oxide (ITO) glasses. The bottom surface was an ITO glass coated with two layers including a photoconductive layer (1 μm a-Si:H) and an adhesion/conductive layer (10 nm molybdenum), as shown in Figure 1(a). The droplet was repelled by the negative ODEP force away from the projected area and attracted to the weaker electric field when a voltage of 60 Vpp was applied at a frequency of 100 kHz. In this study, the electric field and the ODEP force can be calculated by the VTR model and compared with the experiment data reported in our previous work [42]. Figure 1(b) shows the case of the polystyrene beads suspended in DI water with a 1% FBS, which was also manipulated by the negative ODEP force in the same device. The driving voltage and frequency for this case were 36 Vpp and 100 kHz, respectively [48]. Note that the FBS is for reducing the adhesion between bead surfaces and the bottom surface. Similarly, by using the VTR model, the electric field and the ODEP force was investigated and compared with the experiment data reported in our previous work [48].

### 2.3. The CM Factor of the Oil-In-Water Emulsion Droplet and the Polystyrene Bead

This CM factor in Equation (3) is a function of the complex permittivity of the particle and the media, and ranges between 1.0 and −0.5 in this case. Therefore, not only does this factor partially determine the magnitude of the force, but also its direction. In conditions where a particle is more polarizable than the media, the factor is positive and the particle, therefore, experiences a positive DEP force and moves towards regions of higher electric fields. If the particle is less polarizable than the media, the particle moves towards regions of lower electric field since it experiences a negative DEP. Note that in practical applications, the driving frequency and the media conductivity can be adjusted to fine-tune the magnitude and direction of the ODEP forces.

**Figure 2.**
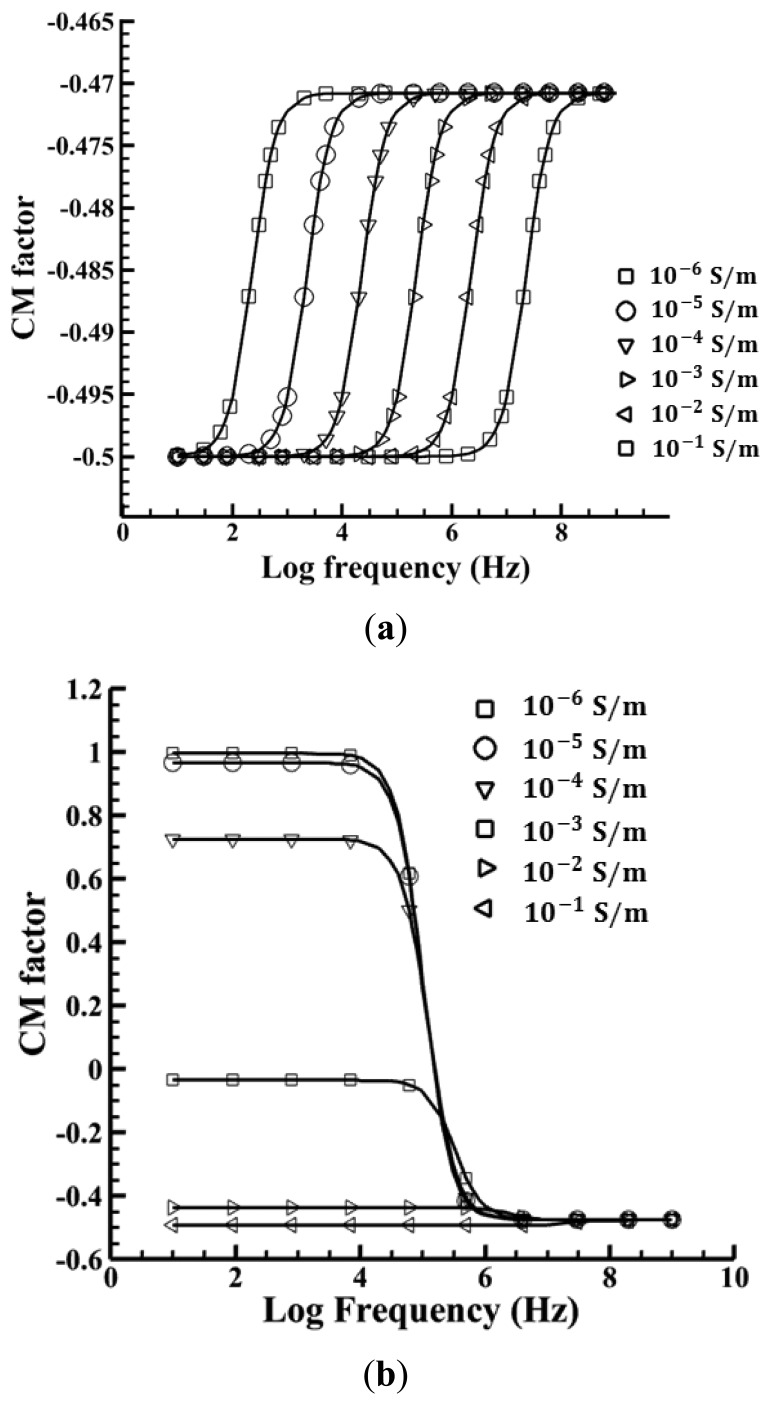
The CM factor of (**a**) oil-in-water emulsion droplets and DI water with 1% Triton X-10 and (**b**) polystyrene beads and DI water with 1% FBS.

Figure 2 shows how the CM factor varies as a function of the driving frequency when the media conductivity ranges from 10^−6^ to 10^−1^ S/m for these two cases. For emulsion droplets suspended in DI water with 1% Triton X-100 surfactant (Octyl Phenol Ethoxylate) (Figure 2(a)), the CM factor is calculated to range from −0.499 to −0.465 even though the media conductivity changes about five orders of magnitude because of the extreme low droplet conductivity. The CM factor in this case is calculated to be −0.499 at a frequency of 100 kHz, where the relative permittivities of corn oil and water are 3.1 and 80, respectively, and the conductivities of corn oil and water are 50 pS/m and 2.3 mS/m, respectively, thus resulting in a negative ODEP force in this case. For polystyrene beads with a 1% FBS (Figure 2(b)), the difference in conductivities dramatically changes the values of the CM factor. At a low conductivity, it is possible to induce a positive DEP (*i.e.*, Re[CM] values greater than 0), where particles are attracted to an electrode edge or other high electric fields. This occurs at low frequencies and transitions to a negative DEP at high frequencies, where particles are repelled into regions with low electric fields. As the conductivity is increased, the strength of the positive DEP force becomes diminished and will eventually turn into a negative DEP force. The CM factor for this case is calculated to be −0.13 at a frequency of 100 kHz, when the relative permittivities of the polystyrene beads and DI water with 1% FBS are 2.5 and 80, respectively, and the conductivities of polystyrene beads and water are 0.9 mS/m and 16.5–17.5 mS/m, respectively. Thus a negative ODEP force is generated in this case as well.

### 2.4. Device Configuration and the VTR Model

**Figure 3.**
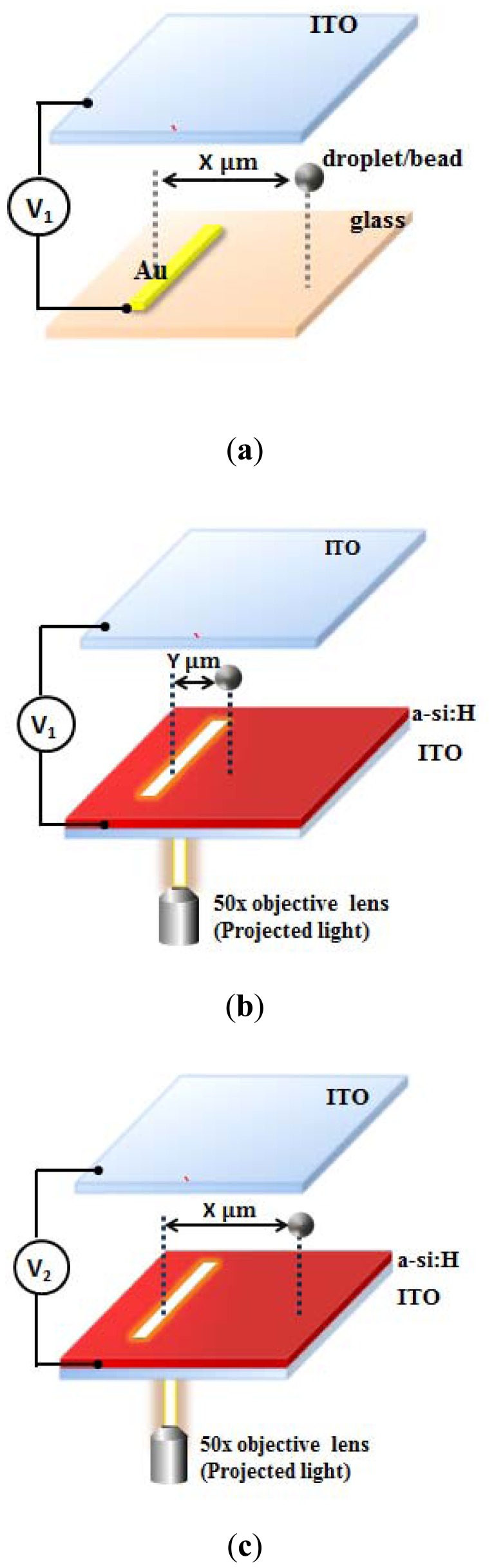
The concept of VTR as illustrated with DEP and ODEP electrodes. (**a**) For the DEP device, an applied voltage (V_1_) repels the droplet to X distance away (**b**) while it only repels the same droplet to Y distance (Y < X)) at the same applied voltage for the ODEP device since a significant voltage drop occurs on the photoconductive layer. (**c**) If the applied voltage is increased to V_2_ (>V1), this results in the same repelled distance (X). V_1_/V_2_ is defined in this manner for the VTR model in this study.

Figure 3 shows a schematic illustration of the DEP and ODEP microdevices and the VTR model by comparing the DEP and ODEP applied voltages (V_1_/V_2_) for simulation of the ODEP force applied on an emulsion droplet. The DEP device was sandwiched between two ITO glasses separated by a 100 μm spacer. The bottom surface was an ITO glass coated with two layers including an electrode layer (gold) and an adhesion layer (chromium). The alternating-current (AC) voltage (V_1_) was applied between the top and bottom layers as shown in Figure 3(a). When an AC bias (V_1_) with a specific driving frequency was applied between two electrodes, non-uniform electric fields were formed in the medium due to the asymmetrical geometry between the top and bottom electrodes, and therefore caused an interaction at the interfaces between the particles and medium and the non-uniform electric fields, resulting in particles moving towards the weakest electric field region.

In the ODEP device, the photoconductive layer was optically excited such that the number of electron-hole pairs increased and the impedance of the amorphous silicon layer decreased when illuminated by light. Consequently, the light-illuminated region could be regarded as the “virtual electrode” which formed a non-uniform electric field. Therefore, the ODEP force was smaller than the DEP force when the same voltage was applied. As shown in Figure 3(b), when the DEP force was applied with V_1_, this caused the droplets to be repelled to X μm away from its original location, while the ODEP force only repelled the same droplet to a smaller distance (Y μm) away. By increasing the voltage to V_2_ to generate the same distance (X μm), as shown in Figure 3(c), the ODEP force could generate the same effect as the DEP force. The VTR (V_1_/V_2_) value to compensate for the photoconductivity effect could thus be characterized experimentally. With this approach, one can obtain the effective driving voltage for the ODEP forces such that the electric field distribution and the resulting ODEP forces could be simulated.

**Figure 4.**
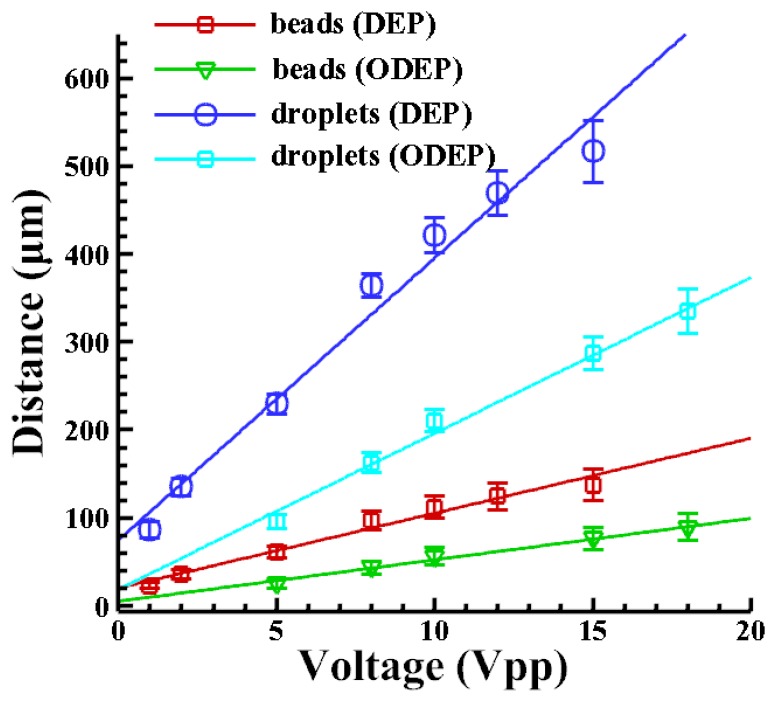
The repelled distance generated by the DEP electrode and the ODEP virtual electrode for a 20 μm droplet. The VTR is calculated as 1/3 in this case when comparing the repelling distance generated by DEP and ODEP forces.

Figure 4 shows the distance that a droplet with a radius of 10 μm is repelled by the DEP and the ODEP forces at different driving voltages. The VTR is calculated accordingly. It shows that the higher the applied voltage; the larger is the repulsive force. The larger force leads to a greater repelled distance; as expected. It is also observed that the DEP-repelled distance is greater than the one caused by the ODEP force. When comparing the repelling distance generated by DEP and ODEP forces; the VTR can be calculated as approximately 1/3 (0.31–0.33) in this case. Similarly; the value is approximately 1/3 (0.30–0.33) in cases with beads. Note that the thickness of the amorphous silicon in our study is 1 μm. Note that the VTR may be highly dependent on the characteristics of the photoconductive materials and their thickness as well.

### 2.5. Experimental Procedure and Fabrication Process

In the DEP device, the fabrication process started with a standard wafer cleaning process in which the glass substrates were immersed in a Piranha solution (H_2_SO_4_(%): H_2_O_2_(%) = 3:1, 120 °C) for 10 min and then rinsed in DI water and blow-dried with nitrogen gas. Using an electron-beam evaporation process, an adhesion layer of 0.02 μm chromium (Cr) was deposited onto the glass substrate, followed by deposition of a 0.2 μm layer of gold. The line-arrays of microelectrodes were then patterned using a standard photolithography and metal etching process. Finally, the photoresist layer was stripped away by an acetone solution and an isopropanol solution, and a patterned Au/Cr electrode was formed [49]. A function generator (Model 195, Wavetek, Stevenage, UK) was used to supply an AC voltage to generate the DEP force. The movement of the droplets/beads were observed under a microscope tube (Zoom 125C, OPTEM, Calgary, Alberta, Canada) and was recorded by a charge-coupled-device camera (SSC-DC80, Sony, Tokyo, Japan) connected to a personal computer.

Alternatively, the ODEP chip was fabricated by first sputtering a layer of ITO onto a glass substrate to form a 70 nm conductive layer. Molybdenum was then sputtered on top of the ITO layer to produce a 10 nm layer that reduced the contact resistance and improved adhesion between the ITO glass and the subsequently deposited amorphous silicon layer. A 1 μm thick amorphous silicon layer (photoconductive layer) was then deposited by using a plasma-enhanced chemical vapor deposition process. The photoconductive layering process was performed by a foundry service (Chi-Mei Optoelctronics Inc., Taipei, Taiwan). A commercial liquid-crystal-display projector (PJ1172, Viewsonic, Chiba, Japan) was used as a light source and was connected to a personal computer to generate the optical images for the virtual electrodes (28 μm-wide line). The light beam was a white light with an intensity of 7.82 W/cm^2^. A 50 × objective lens was used to collect and to collimate the projected light onto the photoconductive layer.

### 2.6. Numerical Simulation of the Electric Field

In this simulation model, the VTR can determine the voltage drop caused by the photoconductive effect so that one can accurately calculate the electric fields. The light propagation can be assumed as a radial Gaussian distribution with a light width of 28 μm, which is the line width used for manipulation of the droplets. There is an electric field gradient in both the lateral and vertical directions, indicating that the generated ODEP forces may pull the nearby particles towards the surface of the virtual electrode if a positive DEP force is induced, or in the opposite direction if a negative DEP force is induced. Specifically, the transformed voltage is exerted by a Gaussian profile due to light propagation. For a Gaussian-distributed beam, the corresponding time-averaged light intensity distribution can be described as follows [50]

(5) $$I=I_{0}\exp\frac{-2{\left(x-x_{0}\right)}^{2}}{r^{2}}$$

where *I*_0_ is the peak value of the light intensity calibrated with the VTR, x_0_ is the transverse location of the peak value and 2r is the width of the light beam as measured between where it decreases by 1/e^2^. A quasi-static electric module which solves the Maxwell’s equation in a commercial program (COMSOL MULTIPHYSICS 3.5A) was used for finite-element simulations of the electric field strength and its distribution in the ODEP device.

**Figure 5.**
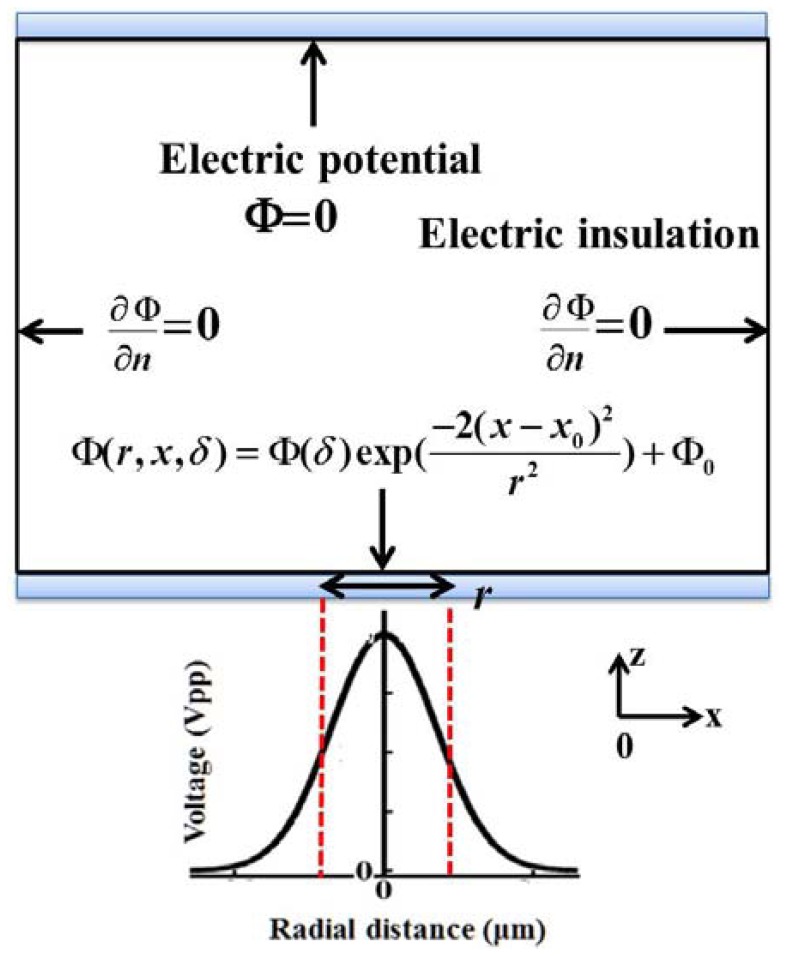
Schematic illustration of boundary conditions for VTR model simulation. Φ(*δ*) is the applied electric potential multiplied with the voltage transformation ratio *δ*.

In this simulation model, the light-patterned boundary condition was an applied voltage with the proposed VTR (δ) while left-hand and right-hand boundaries were insulated (Neumann condition), *i.e.*, *E*_n_ = *E*·*n* = 0 or $\frac{\partial \Phi }{\partial n}=0$, where *n* is the normal vector of the boundary and Φ(*δ*) is applied electric potential multiplied with the VTR. For the upper boundary, it was grounded, as shown in Figure 5.

In addition, the distribution of the transformed voltage must be slightly-adjusted by the initial voltage drop value (when not illuminated), which means that the transformed voltage should be added with an initial value in the simulations. For the case of emulsion droplets, the initial value of the voltage drop is only 2.4 × 10^−2^ Vpp in a 100 μm thick medium layer with a conductivity of 2.3 mS/m when most of the voltage drop occurs in a 1 μm a-Si:H layer with a dark conductivity of 10^−8^ S/m at an applied voltage of 60 Vpp. Note that it is calculated by using the resistance of the medium and the substrate. For the case of polystyrene beads, the initial value of the voltage drop is only 6.5 × 10^−4^ Vpp at an applied voltage of 36 Vpp. Note that the applied voltages for these two cases (60 Vpp and 36 Vpp) has been adopted from the previous studies [42,48].

## 3. Results and Discussion

**Figure 6.**
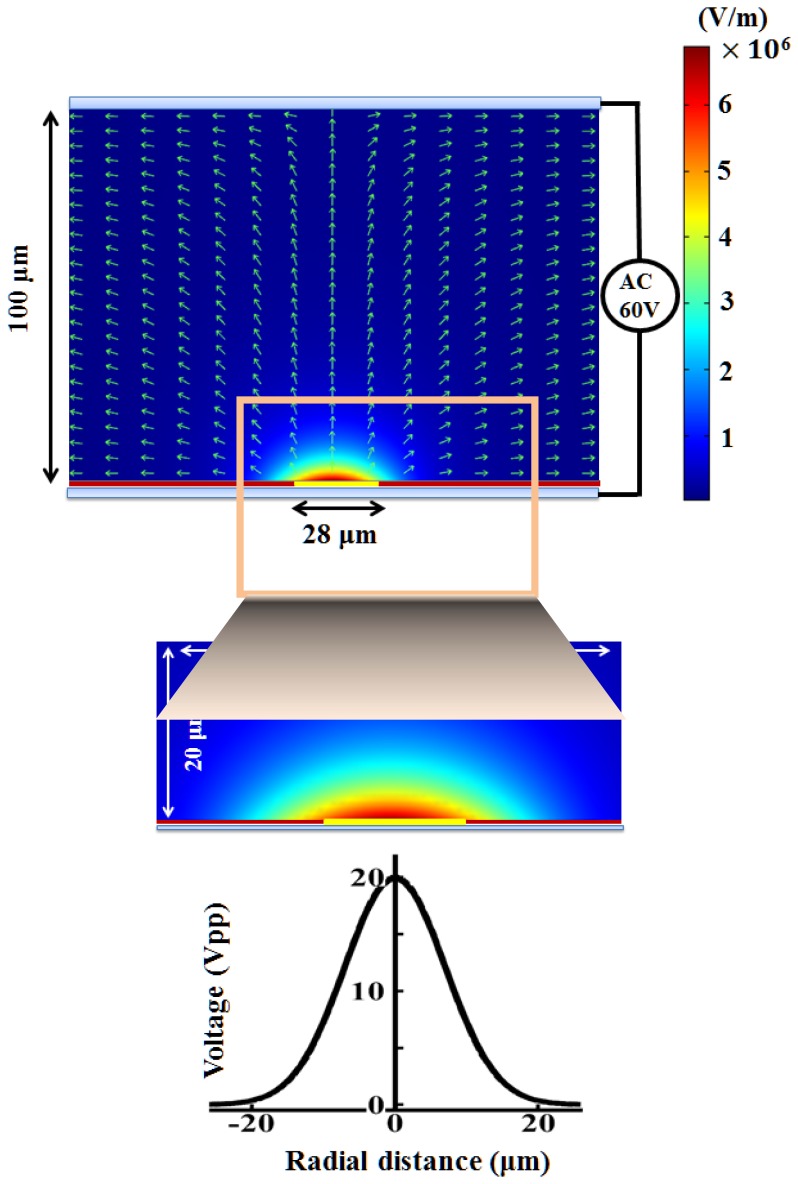
Post-processed visualization of the numerically simulated distributions of the electric field strength inside the ODEP device with the arrows indicating the direction of the ODEP force. The electric field and the profile of the Gaussian distribution of the light pattern for a 20 μm 50 μm enlarged area are characterized.

Figure 6 shows the magnitude of the electric field with the arrows indicating the direction of the ODEP force on the droplets. It is evident that the electric field has a non-uniform profile inside the liquid with the strongest gradient close to the a-Si:H bottom surface. The maximum value of the electric field is calculated to be 6.82 × 10^6^ (V/m). The electric field simulation is performed under the condition that a scanning light beam is projected onto the a-Si:H layer. The original applied voltage is 60 Vpp with a driving frequency of 100 kHz while a VTR value of 1/3 is adopted (20 Vpp). A Gaussian distribution, as described in Equation (5), with a light line width of 28 μm is used. The electric potential in each subdomain is first calculated. Then the electric field strength can be obtained, as presented in Figure 6. The enlarged electric field from the marked region with an area of 20 μm × 50 μm is also shown here. The profile of the Gaussian distribution of the light pattern is also characterized with the largest peak value of the transformed voltage (20 Vpp).

**Figure 7.**
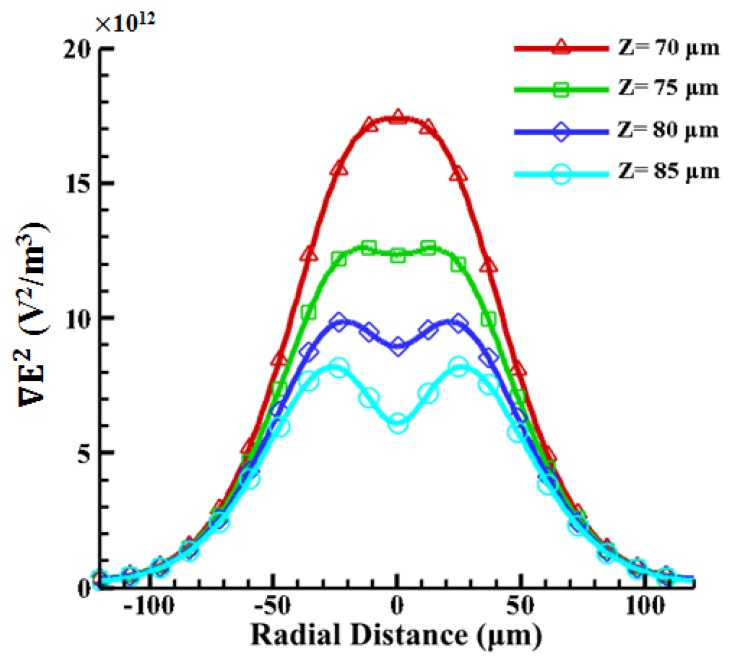
The value of at four different heights (70, 75, 80, 85 μm) as a function of radial distances above the photosensitive surface.

According to the results reported in our previous work [42], the oil-in-water emulsion droplets with a 1% surfactant can be manipulated by using the ODEP force. Since the density of oil is smaller than water and the levitation force from a negative ODEP is induced, it is reasonable to assume a vertical position for the droplets (denoted as “Z”). The radii of the oil droplets are 15, 20, 25, and 30 μm, respectively. Therefore, the corresponding vertical positions are 85, 80, 75, and 70 μm, respectively, in the 100 μm spacer. The magnitude of the ODEP force is proportional to the gradient of the square of the electric field (∇*E*^2^) and can be calculated accordingly. As shown in Figure 7, the magnitude of the electric field gradient at heights of 70–85 μm above the substrate in the horizontal direction generated by ODEP is first calculated. The strongest value of ∇*E*^2^ occurs near the illuminated area on the photoconductive surface. With the increase in the height from the bottom photoconductive layer surface to the top ITO surface, the peak value becomes dramatically smaller, indicating that the ODEP decreases accordingly. Nevertheless, it is found that the central value is lower with the increase in height and, furthermore, changes from one peak to two peaks from 70 to 85 μm. This phenomenon only happens at heights near the upper surface, as shown in Figure 7. This also indicates that the particle can be trapped in the center of the single illuminating light beam as the particle size approaches the width of the light pattern [31].

**Figure 8.**
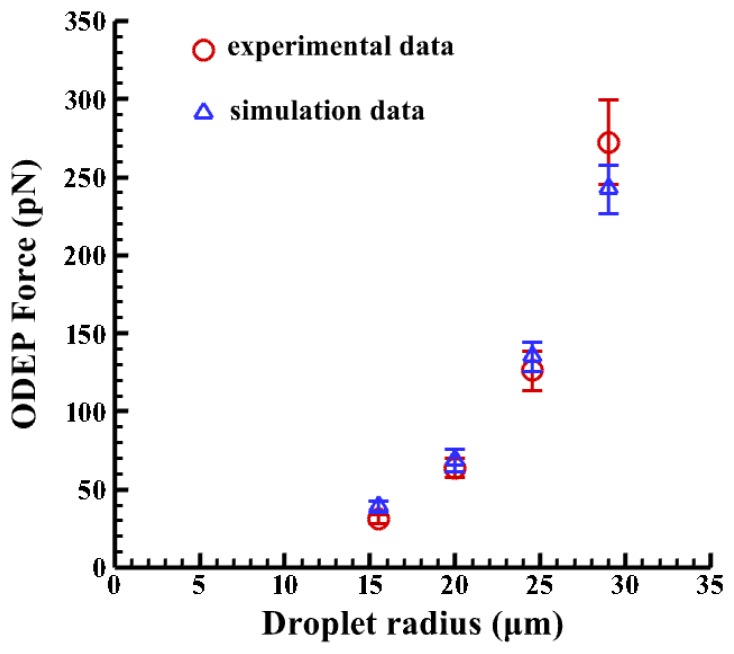
A comparison between the experiment results and simulation data. The emulsion droplet has an applied voltage of 60 Vpp with a driving frequency of 100 kHz in the 100 μm spacer.

Furthermore, the four magnitudes of ∇*E*^2^ are calculated to be 1.68 × 10^13^, 1.25 × 10^13^, 7.32 × 10^12^, 4.57 × 10^12^ (V^2^/m^3^) at the vertical positions (Z) of 70, 75, 80, 85 μm, respectively. Then the ODEP force can be calculated, as shown in Figure 8, when a CM factor of −0.499 is used for droplets. Note that the corresponding horizontal positions are obtained from experimental measurements. It is clearly observed that the bigger the emulsion droplets, the higher the induced ODEP force. The horizontal positions of the droplets are determined by the repelled distances of the droplets, as measured from the experimental results. Oil-in-water droplets for a medium with a conductivity of 23 μs/cm and 1% surfactant (Triton X-100/Octyl Phenol Ethoxylate) experience a ODEP force of 272 pN with a coefficient of variation of 12% (n = 3), as calculated by balancing the fluid drag force [51]. This compares well with the numerical result of 255 pN with a coefficient of variation of 6.2% (n = 3) for a droplet with a radius of 30 μm. The deviation in the numerical results may be due to the variation in the selected horizontal distance of the droplets, as measured from the center of the projected line to the center of the droplets. Thus, the results from the numerical simulation based on the VTR model are in reasonable agreement with the experimental data, with a maximum coefficient of variation of 6.2% (n = 3). Therefore, the proposed VTR model can simulate the generated ODEP force with a reasonable accuracy.

**Figure 9.**
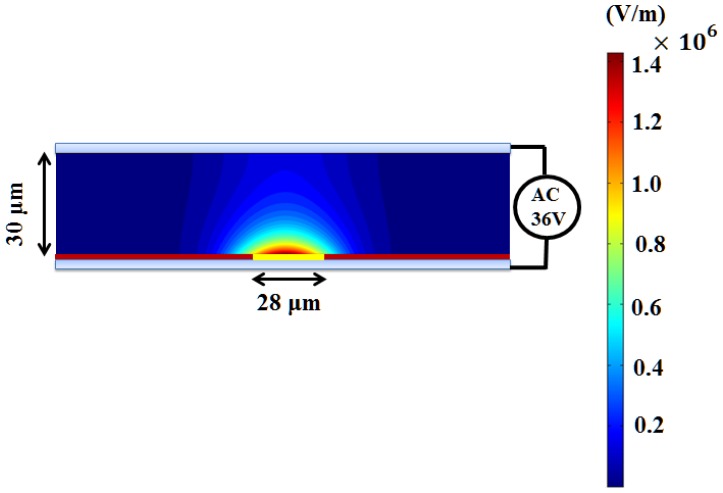
The numerically simulated distributions of the electric field strength inside a 30 μm spacer of an ODEP device.

**Figure 10.**
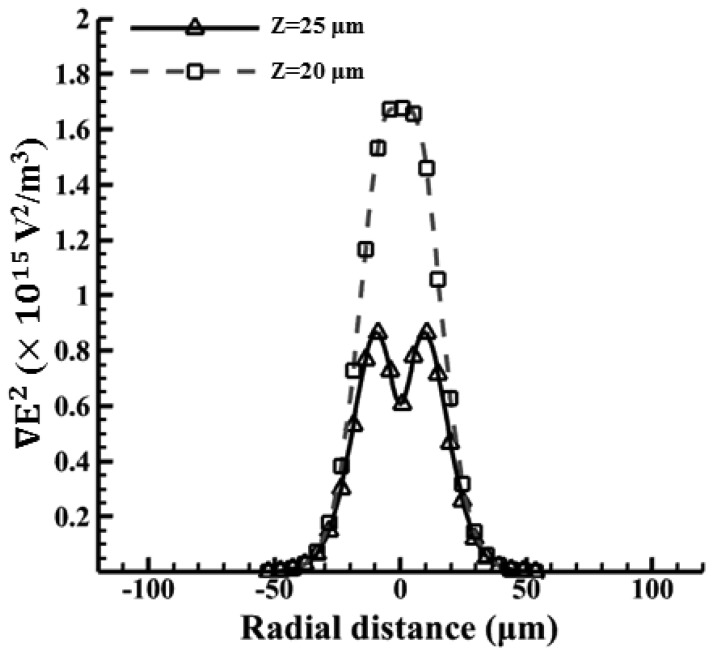
The value of as a function of radial distances above the photosensitive surface at two different heights (20 and 25 μm) for polystyrene beads.

**Figure 11.**
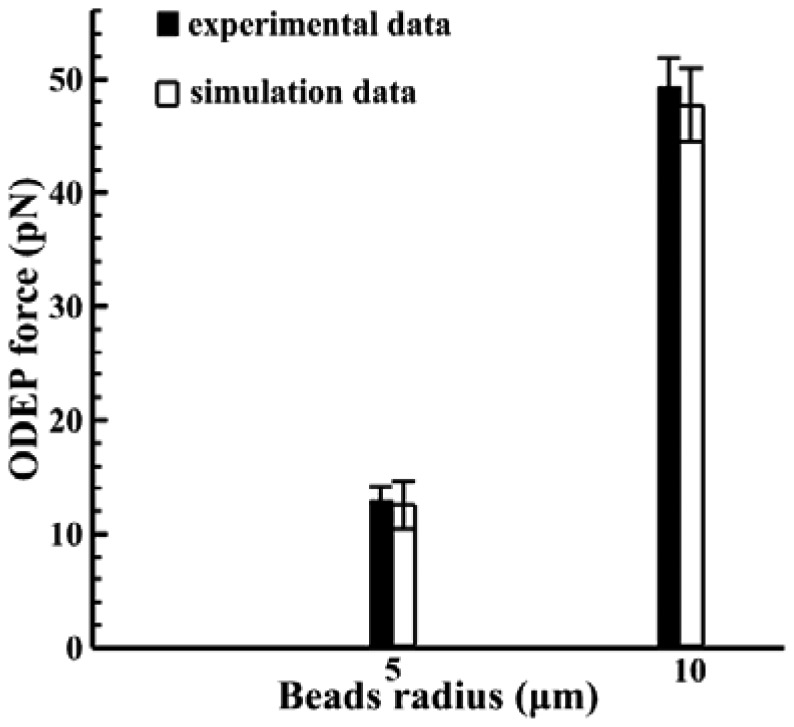
Comparison between experiment results and simulation calculations for polystyrene beads at an applied voltage of 36 Vpp (12 Vpp for the transformed voltage) with a driving frequency of 100 kHz and a 100 μm spacer.

Figure 9 shows the magnitude of the electric field with the arrows indicating the direction of the ODEP force in the cases for beads. The original applied voltage is 36 Vpp with a driving frequency of 100 kHz in this experiment. Note that a VTR value of approximately 1/3 is used (12 Vpp) in this case, as mentioned previously. Then the electric field strength can be simulated accordingly. Note that the maximum value of the electric field is calculated to be 1.4 × 10^6^ (V/m). Similarly, the magnitude of the electric field gradient, at heights of 20 and 25 μm above the substrate, in the horizontal direction are calculated accordingly, as shown in Figure 10. Here, the magnitudes of ∇*E*^2^ are 1.5 × 10^15^ and 4 × 10^14^ (V^2^/m^3^) at the vertical positions (Z) of 20 μm and 25 μm, respectively, which are taken according to their horizontal positions. Therefore, the ODEP force can be calculated accordingly. Note that the horizontal position is obtained from experimental measurements. Figure 11 compares the experimental results [48] and the calculated results for polystyrene beads in a 30 μm spacer. The generated ODEP force for beads with a radius of 5 μm is calculated to be 13.8 pN while the experimental data is 14.5 pN. Note that the coefficient of variation between the experimental data and simulation data is only 4.8% (n = 3). Similarly, the calculated ODEP force for the bigger beads with a radius of 10 μm is 48.5 pN while the experimental data is 50.0 pN. Note that the coefficient of variation between the experimental data and simulation results is 3.0% in this case (n = 3). Again, the numerically simulated results are in reasonable agreement with the experimental data.

In order to increase the electric field and the resulting ODEP force, one can increase the intensity of the light, fine-tune the wavelength of the light, use other photoconductive materials (such as polymers), or optimize the thickness of the photoconductive materials. The proposed VTR model provides a new approach to calculate the ODEP force by measuring the repelled distances in DEP and ODEP devices to calibrate the voltage transformation ratio. This may provide researchers a useful tool for accurately predicting the force on beads, droplets or cells.

## 4. Conclusions

A new VTR model has been proposed which simplifies the voltage drop caused by the photoconductive effect and the resulting ODEP force can be numerically calculated with reasonable accuracy. A Gaussian distribution of the light patterns has been adopted to depict the light propagation on the ODEP electrode edge to distinguish it from physical DEP electrodes. The ODEP forces for emulsion droplets and polystyrene beads have been investigated. The numerically simulated calculations are in reasonable agreement with the experimental results. This proposed new VTR model to simulate an ODEP platform may be promising for designing actuation methods to manipulate micro-objects, including cells, beads, and emulsion droplets.

## Abbreviations

AC: alternating-current
CM: Clausisus-Mossotti
CMOS: complementary metal-oxide-semiconductor
DEP: dielectrophoresis
DI: deionized
FBS: fetal bovine serum
ITO: indium-tin-oxide
ODEP: optically-induced dielectrophoresis
VTR: voltage transformation ratio
a-Si:H: amorphous silicon

**List of symbols**
*E*: electric field strength
E_rms_: root-mean-square value of electric field strength
p: effective dipole moment
ω: angular frequency
F_DEP_: DEP force
I_0_: peak value of the light intensity calibrated with the VTR
I: light intensity distribution
E_n_: normal component of electric field
N: normal unit vector
V_1_: AC voltage applied for the DEP chip to expel particle to x distance
V_2_: AC voltage applied for the ODEP chip to expel particle to x distance
*Z*: height of the droplet
∇*E*^2^: _gradient of the square of the electric field_
*R*: radius of a spherical particle
*ε_m_*: relative permittivity of the surrounding medium
*ε_p_*: relative permittivity of the particle
σ*_p_*: conductivity of the particle
σ*_m_*: conductivity of the medium
Φ: applied electric potential
δ: voltage transformation ratio
